# COVID-19 cases in spectators returning to Finland from UEFA Euro 2020 matches in Saint Petersburg

**DOI:** 10.1017/S0950268822000437

**Published:** 2022-03-18

**Authors:** E. Sarvikivi, M. Salminen, C. Savolainen-Kopra, N. Ikonen, M. Kontio, S. Isosomppi, S. Jamanca, T. Hannila-Handelberg, O. Vapalahti, T. Smura, M. Lappalainen, O. Helve

**Affiliations:** 1 Department of Health Security, Finnish Institute for Health and Welfare, Helsinki, Finland; 2 Epidemiological Operations, Helsinki, Finland; 3 Department of Veterinary Biosciences, University of Helsinki, Helsinki, Finland; 4 Department of Virology, Faculty of Medicine, University of Helsinki, Helsinki, Finland; 5 Department of Virology, University of Helsinki and Helsinki University Hospital, Helsinki, Finland; 6 HUS Diagnostic Center, HUSLAB, Clinical Microbiology, University of Helsinki and Helsinki University Hospital, Helsinki, Finland

**Keywords:** SARS-CoV-2, epidemiology, mass gathering

## Abstract

UEFA Euro 2020 tournament was scheduled to take place in 2020, but due to the coronavirus disease 2019 (COVID-19) pandemic was rescheduled to start on 11 June 2021. Approximately 4500 Finnish spectators participated, travelling between Finland and Russia during the period of 16 to 30 June to attend matches played on 16 and 21 June. A total of 419 persons returning from Russia or with a connection to Russia were detected positive for severe acute respiratory syndrome coronavirus 2 (SARS-CoV-2). Of the 321 sequenced samples 303 turned out to be of the Delta variant. None of these cases was hospitalised. In the following weeks findings of the Delta variant increased rapidly. Thus, EURO 2020 travel-related imported cases likely facilitated this rapid surge of Delta variant, but this impact would likely have been seen with the typical increase in the number of travellers entering Finland later in the summer.

## Background

**Fig. 1.**
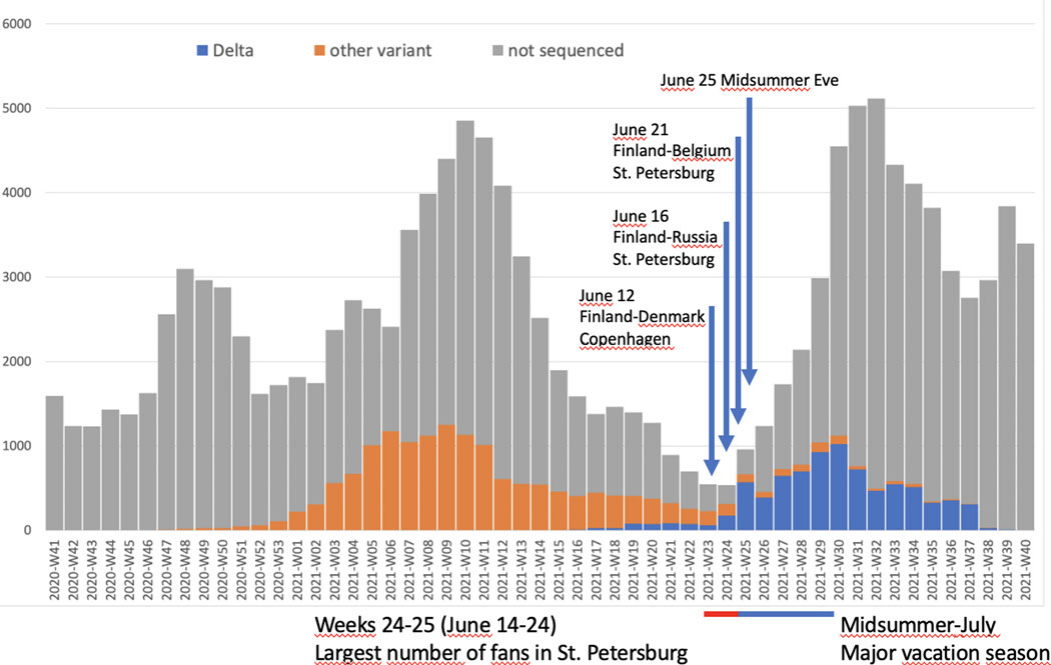
Weekly number of confirmed cases of SARS-CoV-2 and the proportion of delta variant and other lineages in Finland from week 41, 2020 to week 40, 2021. Timing of the Finnish National Football Team's matches in EURO 2020, and the timing of Midsummer eve, are indicated by arrows.

The Finnish National team had qualified for the first time for the EURO championships, and the general interest towards the games was substantial, both in 2020 and, after postponement, in 2021. Many were determined to attend the matches, as severe acute respiratory syndrome coronavirus 2 (SARS-CoV-2) case numbers were dwindling in Europe and also in Finland in the early summer of 2021 (Fig. 1). However, the epidemic situation began to deteriorate in St. Petersburg in June.

The Finnish team's matches in St. Petersburg took place on 16 June and 21 June. As the epidemic situation in Russia worsened, The Finnish Institute for Health and Welfare (THL) discussed the potential travelling from Finland to St. Petersburg with the Ministry of Health and the Ministry of Traffic and Communication. The train service between Helsinki and St. Petersburg had already been discontinued due to the pandemic and was not initiated for the tournament. The majority of spectators were expected to cross the border by cars and buses. Therefore, THL contacted the Football Association of Finland and the fan club of the Finnish team, as well as bus companies, to distribute information on infection control to potential spectators. THL broadcasted repeatedly a national recommendation for watching the games from home rather than travelling abroad.

Border control checkpoints existed both ways for the spectators. On the Russian border, a certificate of a negative test taken within 72 h was required when entering Russia. Russian border officials also performed extra testing on those with a negative test certificate upon entry, and some spectators with a certificate were turned back due to a positive border test. When returning to Finland, on the Finnish border, a certificate on a negative test taken within 72 h or testing at the border was required. Local healthcare staff was responsible for the border testing. After entry, returning travellers were advised to self-isolate until they had been tested negative the second time (at 72 h after entry at the earliest).

We aimed at quantifying the number of coronavirus disease 2019 (COVID-19) cases in returning spectators to assess the impact of these cases on the spread of the at the time relatively rare Delta variant in Finland [1].

## Materials and methods

We identified all SARS-CoV-2 cases notified to the National Infectious Disease Register (NIDR) during 16–30 June 2021. Those with a known link to Russia based on the notification were included in the study. The vaccination status of these individuals was obtained by a linkage between the NIDR and the National Vaccination Registry, utilizing the unique personal identifiers of the cases.

Whole-genome sequencing of the SARS-CoV-2 samples was performed using the ARTIC protocol (https://artic.network/ncov-2019,) The samples were sequenced with Illumina Novaseq sequencer. Adapter, low quality (quality score <30) and short (<50 nt) sequences were removed using Trimmomatic [2], followed by assembly using BWA-MEM [3], variant calling using LoFreq [4] and consensus calling using SAMtools [5] implemented in HaVoC pipeline [6], followed by lineage annotation with Nextclade (clades.nextstrain.org) and Pangolin (https://cov-lineages.org/resources/pangolin.html). GISAID Accession IDs are shown in (Table 1). We aligned sequences using MAFFT (multiple alignments using fast Fourier transform; https://mafft.cbrc.jp) and constructed the phylogenetic tree with IQ-TREE 2 (http://www.iqtree.org) using ModelFinder [7] for nucleotide substitution model selection, 1000 ultrafast bootstraps and least square dating (LSD2) method [8].

**Table 1.** GISAID Accession IDs of the Delta variant sequences

EPI_ISL_3020916
EPI_ISL_3020960
EPI_ISL_3020965
EPI_ISL_3020967
EPI_ISL_3020972
EPI_ISL_3020977
EPI_ISL_3020978
EPI_ISL_3020981
EPI_ISL_3134984
EPI_ISL_3135022
EPI_ISL_3135029
EPI_ISL_3135052
EPI_ISL_3135064
EPI_ISL_3135093
EPI_ISL_3135094
EPI_ISL_3135095
EPI_ISL_3135122
EPI_ISL_3135743
EPI_ISL_3135762
EPI_ISL_3135765
EPI_ISL_3135768
EPI_ISL_3135784
EPI_ISL_3135790
EPI_ISL_3135791
EPI_ISL_3135800
EPI_ISL_3135805
EPI_ISL_3135807
EPI_ISL_3135812
EPI_ISL_3135851
EPI_ISL_3230447
EPI_ISL_3230448
EPI_ISL_3230450
EPI_ISL_3230452
EPI_ISL_3230453
EPI_ISL_3230458
EPI_ISL_3230497
EPI_ISL_3230506
EPI_ISL_3230507
EPI_ISL_3230508
EPI_ISL_3230537
EPI_ISL_3230538
EPI_ISL_3230598

Data were also gathered by direct communication with the unit responsible for infection control in each hospital district in Finland. THL requested from regional operators daily reports of all cases with a link to EURO 2021 tourism identified. Immediately after the first cases were notified, the reporting was done by email or telephone. Data were collected by a web-based survey between 29 June and 9 July 2021.

## Results

Approximately 4500 spectators travelled to St Petersburg to watch the games on 16 June and 21 June. They travelled by bus, minibus, car and even by bicycle, only a small number flew to St Petersburg. Most of the returning traffic from St. Petersburg to Finland took place between 21 and 25 June. Two border control land checkpoints were used by those returning from St Petersburg. On 22 June, the Vaalimaa checkpoint on the Finnish side became severely congested, and approximately 800 returnees were allowed to enter without checking for a negative test certificate or being directed to a border test. All those were informed to self-isolate and perform a test at 72 h after entry to Finland at the earliest.

**Fig. 2.**
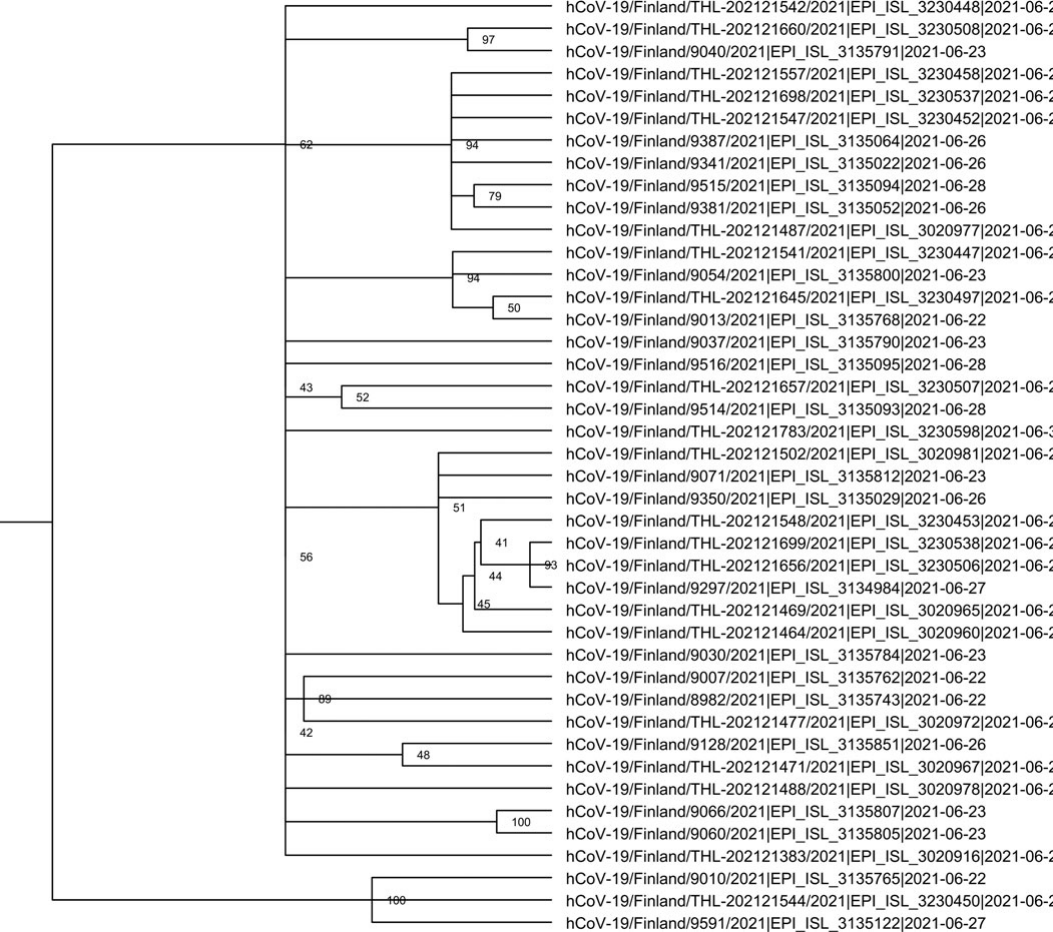
Phylogenetic analysis of the Delta variant cases.

During the period of 16 to 30 June 2021 a total of 419 persons returning from Russia or with a connection to Russia were detected positive for SARS-CoV-2. Out of these, 321 (77%) specimens were sequenced in order to determine the genetic type. The majority of these (303, 94%) turned out to be Delta variants. Alpha variant was detected in one and wild type in two cases. The sequencing result was indeterminate or ambiguous in 15 cases (5%). Out of those carrying the Delta variant, 42 had a known connection to the UEFA Euro 2020 tournament in St Petersburg. However, it is likely that a majority of cases detected among those crossing the border from Russia between 16 and 30 June and carrying the Delta variant were football fans. Sequence and phylogenetic analysis support this notion, but the Delta variants do form different clusters suggesting different places of exposure in St. Petersburg (Fig. 2). Based on sequence and phylogenetic analysis, the Delta variants form different clusters indicated different origins of strains (Fig. 2)

None of the cases with confirmed Delta variant with a connection to UEFA Euro 2020 was hospitalised. 16/42 cases (38%) had received one dose of vaccination (more than 14 days between vaccination and sampling) and one (2%) had received two vaccine doses.

According to the data obtained from the regions, between 16 June and 9 July, there were 501 primary cases among tournament spectators, and 188 secondary cases linked to them. The cases were identified mostly in the capital area, but also in Tampere, Turku, and several other cities across Finland. These cases accounted for 17% of all confirmed cases of SARS-CoV-2 during this time period. Later estimates indicate that 10–15% of fans got infected during their stay in St. Petersburg or on the return travel.

## Discussion

Travel-related importation of SARS-CoV-2 enabled the virus to spread very quickly across the world during the first weeks and months of 2020 [9]. Controlling this spread proved to be very challenging. In June 2021, the epidemic seemed to be under control in Finland. In addition, especially young adults were suffering from severe pandemic fatigue at the time, and these issues probably encouraged the fans to attend the EURO 2020 on site.

Several pitfalls in infection control during travel were identified. Control measures such as passenger volume and face mask use in buses had been insufficient. Passenger lists were often lacking, and passengers also had changed seats and vehicles during the trip. Thus, the average number of contacts during travel had increased, and also effective contact tracing was challenging. The only possible way to reach the travellers was through the companies that had offered transportation to St. Petersburg for the spectators. Unclear responsibilities between officials, travel and transport companies, and travellers contributed to case surges.

Almost in all the buses returning on week 25, when passengers were screened, positive cases were found. Therefore, all people that were known to have travelled from Russia to Finland by bus during week 25, were placed in quarantine on 28 June, by the local infection control units, according to guidance by THL.

The third wave of the SARS-CoV-2 epidemic in Scotland is considered to have been impacted by spectators returning from the EURO 2020 tournament in London [10]. The fan-travel for EURO 2020 was not responsible for the introduction of the Delta variant in Finland, as the virus had been introduced more than a month earlier (Fig. 1), and community transmission had been established before this incident. The fan travel was not either solely responsible for the surge in cases seen in Finland after the tournament, but the surge of COVID-19 cases had started prior to the return of the fans and was impacted by the fact that almost all substantial restrictions of socializing had been lifted by the start of June in Finland. However, the timing of the travel-related additional cases was challenging, as most fans returned right before Midsummer weekend. Midsummer is traditionally spent with family and friends gathering for private parties with multiple contacts, which enabled effective spread. Also, the resources for contact tracing were scarce, and as no extra personnel was available, strengthening the contact tracing teams was not possible. Multiple new transmission chains started during the Midsummer weekend, and this very likely aggravated the epidemic situation in Finland.

It is likely that the several hundred imported primary cases spread the virus effectively, especially due to the timing of introduction overlapping with national festivities associated with Midsummer. In addition, the previous situation with declining numbers of cases and general confidence of the protective effect of vaccines may have contributed, as people were likely to be less concerned about the pandemic. However, this impact would likely have been seen with the typical increase in the number of travellers entering Finland later in the summer. Therefore, it is likely that the EURO 2020 did not have a major impact on the overall epidemic situation in Finland. It is unlikely that interventions, such as strict recommendations against group gatherings during Midsummer festivities or more centralised organisation of travelling resulting in more fluent border control measures, would have changed the eventual outcome. However, EURO 2020 related imported cases likely facilitated the surge of Delta variant in Finland.

## Data Availability

The data on the COVID-19 cases in Finland and the number of tests as reported by the laboratories performing such tests are openly available in THL Open Data at https://thl.fi/en/web/thlfi-en/statistics-and-data/data-and-services/open-data/confirmed-corona-cases-in-finland-covid-19-
